# Sensitivity to trustworthiness cues in own- and other-race faces: The role of spatial frequency information

**DOI:** 10.1371/journal.pone.0272256

**Published:** 2022-09-06

**Authors:** Valentina Silvestri, Martina Arioli, Elisa Baccolo, Viola Macchi Cassia

**Affiliations:** Department of Psychology, University of Milan-Bicocca, Milan, Italy; PLOS, UNITED KINGDOM

## Abstract

Research has shown that adults are better at processing faces of the most represented ethnic group in their social environment compared to faces from other ethnicities, and that they rely more on holistic/configural information for identity discrimination in own-race than other-race faces. Here, we applied a spatial filtering approach to the investigation of trustworthiness perception to explore whether the information on which trustworthiness judgments are based differs according to face race. European participants (N = 165) performed an online-delivered pairwise preference task in which they were asked to select the face they would trust more within pairs randomly selected from validated White and Asian broad spectrum, low-pass filter and high-pass filter trustworthiness continua. Results confirmed earlier demonstrations that trustworthiness perception generalizes across face ethnicity, but discrimination of trustworthiness intensity relied more heavily on the LSF content of the images for own-race faces compared to other-race faces. Results are discussed in light of previous work on emotion discrimination and the hypothesis of overlapping perceptual mechanisms subtending social perception of faces.

## Introduction

Our understanding of the social world is highly dependent on the rapid and automatic evaluation we make of our conspecifics based on their facial appearance. Faces are complex and highly dynamic stimuli conveying a wide variety of social cues that we can effortlessly extrapolate and use to consequently adapt our social behavior. Perception of invariant aspects of faces allows us to categorize other people’s ethnicity or gender and recognize their identities, while perception of more changeable aspects of faces, such as eye gaze or expressions, allows us to infer other people’s emotional status, mental state or intention, which is particularly important for social communication.

One fundamental component of our social competence is our ability to rapidly and spontaneously infer others’ intentions towards us, that is whether they are likely to approach us friendly or hostilely, and thus whether we can trust them or better not. The facial configuration on which we draw such inferences is known as facial *trustworthiness* [1]. Trustworthiness judgments are known to be fast, automatic, based on very little information and performed with high consensus [2, 3]. These fast and automatic responses are thought to derive from evolutionary pressure that has taught us to pay attention to those facial cues signaling potential harm, and to distinguish between friends or foes to enhance our chances of survival [4]. Not by chance, humans manifest a precocious sense for threat, which affects their behavior and attentional responses from the earliest stages of development [5]. Accordingly, by the age of 7 months infants show behavioral and electrophysiological signatures of sensitivity to facial signs of trustworthiness [6–9].

The hypothesis that sensitivity to facial cues to trustworthiness is somehow rooted in our phylogeny also finds support in the results of the few cross-cultural studies conducted on the topic, in which trustworthiness judgments do not appear to be modulated by the perceiver’s cultural background [9–11]. Xu and colleagues [11] showed that Chinese and European adults used similar facial information to generate explicit trustworthiness judgments from White faces, as they both relied on facial attractiveness, inner brow ridge or skin shade. Similarly, Birkás et al. [10] reported that European and Asian adults generalize their perception of trustworthiness intensity to faces of different ethnicities (European, African, South Asian and East Asian). Sakuta and colleagues [9] even reported an attentional preference for trustworthy over untrustworthy White faces in preverbal Japanese infants, suggesting again that perception of trustworthiness is a cross-cultural phenomenon, and that facial cues to trustworthiness are somewhat universal.

Nonetheless, evidence from these same studies also showed that trustworthiness perception is to some extent modulated by an individual’s expertise with own-race faces. For example, in the study by Birkás et al. [10] European and European American participants judged White faces as overall more trustworthy than other-race faces, and Sofer and colleagues [12] showed that European adults perceived own-race typical faces as more trustworthy than other-race typical faces, suggesting that people in both cultures employ typicality cues when judging trustworthiness, but that these cues are culture dependent. Thus, although limited, available evidence suggests that sensitivity to facial cues to trustworthiness lies at the intersection of biological and experiential mechanisms, where innate sensitivity to faces and others’ behavior facilitate and canalize perceptual and cultural learning through ontogenetic experience.

Indeed, in a recent account of the origins of spontaneous face-traits inferences, Over and Cook [13] propose that, during ontogeny and through associative learning, we become able to map the representation of individuals’ facial appearance comprised in our face space (i.e., the multidimensional space within which the visual system represents the faces it encounters [14]) to the conceptual knowledge of others’ personality traits as represented in our trait space (i.e., the multidimensional space within which we represent stable characteristics of an individual that predict their likely responses and behaviours across a range of situations) [13]. Within this view, experience plays a critical role in shaping face-traits inferences, as both our face perception ability and our knowledge of other people’s traits are subject to considerable environmental control.

The face-processing literature provides many demonstrations of how experience within our social environment shapes face perception abilities, giving rise to a number of so-called *face processing biases* [15]. The most well-known example of this is the *own-race bias* [14, 16], a reliable phenomenon across cultural and ethnic groups where faces belonging to ethnicities that are underrepresented in the individual’s social environment are recognized with greater difficulty with respect to those that are more frequently experienced. This phenomenon has been interpreted within an integrative framework where both social cognitive variables related to the in-group or out-group status of the face and perceptual expertise derived from intergroup contact interact as they both contribute to an individual’s ability to focus on diagnostic facial features [17, 18]. In particular, research in both adult and developmental populations has shown that environmental exposure to own-race faces during ontogeny leads to enhanced sensitivity to the featural and configural cues that differentiate individual faces from the over-experienced category [19, 20] and the construction of a well-refined representation of such category in face memory [21].

In light of this, it is reasonable to expect that perceptual tuning towards own-race faces also translated in differential sensitivity to physical cues to trustworthiness in own-race versus other-race faces. A viable way to explore this question is to examine the nature of the visual information that is necessary to elicit trustworthiness judgments from own-race and other-race faces by manipulating the spatial frequencies (SF) content of the stimuli corresponding to featural or configural information. A large number of studies have supported the associations between high spatial frequencies (HSF) and the processing of the fine-scale local details of the original face image and between low spatial frequencies (LSF) and the processing of the large-scale global shape of the face [22, 23]. On this basis, spatial filtering (i.e., the selective removal of portions of the SF information contained in the image) can be used to assess the contribution of local and/or global information to participants’ performance in any face-processing task.

This approach has been extensively used to explore what information we extract from faces when we discriminate emotional expressions [3, 24, 25]. In adults, emotions modulate Event Related brain Potentials (ERPs) when faces contain lower spatial frequencies [24], and LSF information is crucial to produce an increase in fMRI activation to fearful faces relative to neutral faces in the amygdala, a key subcortical structure in emotional processing, as well as in the visual cortical areas [25]. Differential sensitivity to HSF and LSF contents of emotional expressions has also been found in some behavioral tasks: emotion categorization [26] and attentional responses to fear [27] occur more rapidly for LSF faces than for HSF faces, whereas participants’ ratings of fear intensity increase in the presence of HSF information [25]. It is claimed that distinct streams in the visual system of primates are selectively sensitive to different ranges of spatial frequencies [28, 29]. On this basis, these and other results from similar studies have been taken as indicative that the encoding of emotional facial cues relies mainly on the activation of the magnocellular visual pathway that provides rapid, but coarse, visual signals to the amygdala, and then to the visual cortex feeding into the dorsal stream [25].

Although much research has been conducted to establish which distinct facial pieces of information evokes trustworthiness perception in the perceiver [30, 31], only few studies have investigated how the spatial resolution in which such pieces of information are visually processed impacts trustworthiness ratings [32, 33]. These studies indicated that the eye and eyebrow region modulates trustworthiness judgments when their HSF components are available, while the mouth plays a role when revealed in its LSF components [33].

In the current study, we applied the spatial filtering approach to the investigation of trustworthiness perception to explore whether the information on which trustworthiness judgments are based differs according to participants’ expertise with specific face types. To the best of our knowledge, so far only two studies adopted this same approach [32, 34]. The first one tested for the modulatory effects of image spatial frequency filtering on the amygdala response to very trustworthy and very untrustworthy faces using fMRI [34]. In contrast with the authors’ predictions based on previous work on emotional expressions [25], neuroimaging results showed comparable amygdala sensitivity to variations in trustworthy intensity in LSF and HSF images, suggesting that trustworthiness information is carried by both coarse and fine resolution visual signals. The authors also recorded behavioral responses by asking participants to rate the faces on perceived trustworthiness, and obtained mixed results. Consistent with the amygdala neuroimaging results, for participants who provided their responses inside the scanner, ratings made on both HSF and LSF faces significantly correlated with ratings for the original unfiltered faces. However, consistent with previous work on emotion discrimination [3], for a second group of participants tested outside the scanner, ratings for the unfiltered face images correlated better with LSF ratings than with HSF ratings.

A second, more recent study manipulated trustworthiness intensity (high, neutral, low trustworthiness) and the coarse versus fine-grained information and SF bands available in White and Black faces by means of the Bubbles method [35] to pinpoint the perceptual information used by European participants to successfully decide which of two faces was the most trustworthy [32]. Results showed that, although similar facial features were used to rate trustworthiness intensity of own- and other-race faces, individuals with stronger implicit racial biases relied more on LSF information in their rating of the former than the latter faces.

In light of these conflicting results and the scarcity of studies on trustworthiness perception across face ethnicity, the current study had three main aims: (a) to explore the effects of high-pass and low-pass spatial filtering on trustworthiness discrimination, (b) to test whether trustworthiness discrimination generalizes across face ethnicity, and (c) to investigate whether coarse and fine resolution information contribute differently to trustworthiness perception in own-race and other-race faces. Based on previous demonstrations that trustworthiness judgments approximate the valence evaluation of faces [36], we predicted greater sensitivity to differences in trustworthiness intensity for LSF images compared with HSF images. However, based on evidence of greater holistic/configural processing for own-race than other-race faces in identity discrimination tasks [37], we also expected to observe greater contribution of LSF information to trustworthiness discrimination of own-race faces compared to other-race faces.

Participants’ sensitivity to differences in trustworthiness intensity was inferred from their performance in an online version of a *Pairwise Preference task* used in previous studies on trustworthiness perception in adults and children [38]. Participants were asked to indicate which face they would trust more within a pair randomly selected from a five-step trustworthiness continuum, and the participant’s response was used to compute a trustworthiness score for each face of the continuum.

## Methods

### Participants

Online data collection took place between April and June 2020. One-hundred eighty-five participants were tested, and 20 were excluded from the final sample as they did not finish the experiment. The final sample thus included 165 young adult participants (125 women; M age = 23 years; range = 18–35 years); all were of European descent, had normal or corrected-to-normal vision, were right-handed and without history of any psychiatric or neurological disorders.

To allow estimation of the minimum sample size in future studies based on current results, using the Simr package in R [39] we calculated that the smallest effect size we could have obtained for the target Spatial filtering x Face ethnicity interaction on our accuracy data with a power > 80% is .33, and the smallest effect size we could have obtained for the target Spatial filtering x Face ethnicity x Trustworthiness intensity interaction on the trustworthiness scores with a power > 80% is .41. Participants were either undergraduate or graduate university students receiving course credits or recruited from the community by word of mouth on a voluntary basis, and they gave their informed consent before completing the task. The protocol was carried out in accordance with the ethical standards of the Declaration of Helsinki and was approved by the Ethics Committee of the University of Milano-Bicocca.

### Stimuli

**Fig 1:**
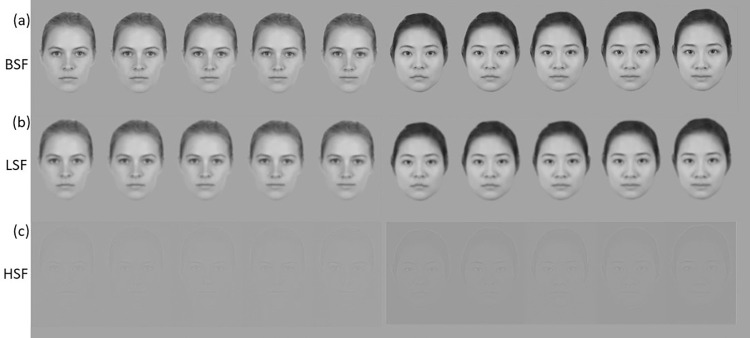
Stimuli used in the present study. The original unfiltered images of the five variations of the female White identity and the female Asian identity used as stimulus material in the Broad-Spatial-Frequency (BSF) condition, representing two trustworthiness continua ranging from 1 (very untrustworthy) to 5 (very trustworthy) (a). The White and Asian Low-Spatial-Frequency (LSF) (b) and High-Spatial-Frequency (HSF) (c) trustworthiness continua generated by filtering the original images.

Stimuli were realistic grayscale Low-Spatial-Frequency (LSF), High-Spatial-Frequency (HSF), and Broad-Spatial-Frequency (BSF) images of five variations of one White female face identity and five variations of one Asian female face identity. For both ethnicities, the five variations reflected a continuum of trustworthiness that ranged from 1 (very untrustworthy) to 5 (very trustworthy), interleaved by a neutral face (Fig 1). The White continuum was obtained by selecting a subset of 5 faces (i.e. by excluding the two trustworthiness extremes) from a previously validated seven-step continuum [38]. The Asian continuum was generated following the same procedure adopted to create the White stimuli (see details in Baccolo & Macchi Cassia, [38])—i.e. by morphing an averaged neutral face toward an averaged untrustworthy and an averaged trustworthy face using an online program for image transformation [40]. The averaged trustworthy and untrustworthy faces were created by merging three different face identities taken from by the Chicago Face database [41], in which faces are rated on the trustworthiness dimension; the averaged neutral face was obtained by merging four neutral identities taken from the same database. The averaged neutral face was morphed towards the trustworthy and untrustworthy averaged references obtained from the three most trustworthy and the three most untrustworthy White or Asian identities available in the Database by two steps (30% and 60%), thus obtaining a five-step trustworthiness continuum which included the neutral face.

To create the LSF and the HSF versions of each face, a spatial frequency filter was applied to each image using a Matlab script available online at https://psy.van-diepen.com/pvdmatl.html (see Dobkins and Harms, [42]). For LSF images, the filtering was done around a frequency cutoff of 1.6 cycles/° (or 24 cycles/face width), so that these images contained frequencies primarily below 1.6 cycles/°. HSF images were similarly filtered with a high-pass cutoff of 2.4 cycles/° (or 36 cycles/face width); cut-off values were chosen after Dobkins and Harms [42]. The unfiltered original images were used as BSF stimuli (Fig 1).

All images measured 12 cm x 8 cm. Participants were instructed to sit at 30 cm from the computer screen, so that each image subtended approximately 22° of visual angle vertically and 15° horizontally. As in other studies exploring the impact of spatial frequency filtering on face processing [43–45] the BSF, LSF and HSF images differed in terms of Root Mean Square (RMS) (BSF: 8.91 cd/m^2^; LSF: 8.78 cd/m^2^; HSF: 2.67 cd/m^2^) contrast, a commonly used index for perceived contrast in natural images. Contrarily, the BSF, LSF and HSF images did not differ in luminance (*p* > 0.9, as calculated based on the RGB values using Matlab), as in other studies using the same filtering procedure [42, 46].

### Stimuli validation

Details of the validation procedure for the original seven-step continuum from which the five White faces were taken is described in full in Baccolo & Macchi Cassia [38]. Here we ran a further validation study to ensure that the five selected White faces and the five newly created Asian faces actually reflected a continuum of expressed trustworthiness. To this end, we asked an independent sample of 53 European Italian adults (40 women; Mage = 22.45 years; range = 19–30) to rate perceived trustworthiness for each White and Asian BSF face on a scale ranging from 1 (I wouldn’t trust this person at all) to 9 (I would definitely trust this person). Face ethnicity was blocked across trials, and the order in which trial blocks were presented was counterbalanced across participants. Participants provided their ratings through an online Questionnaire delivered on Qualtrics (Qualtrics, Provo, Utah, USA, https://www.qualtrics.com). Adults’ ratings were entered into a repeated-measures ANOVA with trustworthiness intensity (1, 2, 3, 4, 5) and face ethnicity (White, Asian) as within-subjects factors. The analysis revealed a main effect of trustworthiness intensity, *F*(4,208) = 16.99, *p* < .001, *pη*^2^ = .25; a test of within-subjects contrasts revealed a significant linear trend, *t* (208) = 7.91, p < .001, CI lower = .56, CI upper = .93, indicating that the higher the position along the trustworthiness continuum, the higher the trustworthiness scores attributed by the participants. No significant effects involving face ethnicity were observed.

### Apparatus and procedure

The experiment took place online, and participants provided their responses using Qualtrics (Qualtrics, Provo, Utah, USA, https://www.qualtrics.com). They all ran the experiment on a desktop or portable computer, and were instructed to sit approximately 30 cm from the monitor, which, for all participants, was 13 inch or larger with a resolution of at least 1280 × 800 pixels. Participants performed a *Pairwise Preference task* (see Baccolo & Macchi Cassia, [38]) where, on each trial, two faces randomly selected from the same trustworthiness continuum simultaneously appeared on the screen, and they were asked to select the face they trusted more by clicking the mouse. Subjects were told to respond by “using their gut”. They started each trial by pressing the mouse, and the stimuli remained on the screen until a response was made. The task consisted of a 2 (face ethnicity: White, Asian) x 3 (spatial filtering: LSF, HSF, BSF) conditions design. Trials from each of the six conditions were presented in blocks, and participants completed 10 trials per condition, corresponding to all possible pairwise combinations of the five faces of the continuum (60 trials in total). Each face of the continuum was compared to all other faces of the same continuum for a total of four times; the position of the faces on the screen was randomized across trials, and trials within each block were presented in random order. The order of the spatial filtering blocks was fixed across participants: they all completed the LSF blocks first, followed by the HSF blocks and then the BSF blocks. Face ethnicity was alternated between blocks, with the ethnicity of faces in the first block counterbalanced across participants.

## Results

### Response accuracy

One-sample *t* tests (vs. 50%) on percent accuracy, that is the percentage of trials in which the face displaying more intense cues to trustworthiness was selected, confirmed that, for both White, *p*s < .001, and Asian faces, *p*s < .001, participants systematically selected the more trustworthy face in the pair under all spatial filtering conditions. To explore how SF filtering affected the likelihood that participants selected the more trustworthy face in the pair participants’ response accuracy on each single trial of each condition, expressed as 0–1 scores (0 if the selected face was the less trustworthy in the pair, 1 if it was the more trustworthy one) was entered as dependent variable into generalized mixed-effects logistic regression models. The analysis was performed using the GAMLj 2.3.0 module in the Jamovi software (Version.1.6.15; The jamovi project, 2021). Following the recommendations of Meteyard and Davies [47], the models’ parameters selection was driven by the research question, and so we only included relevant interactions, which also helps avoiding overfitting issues. Specifically, we included face ethnicity (White, Asian) and spatial filtering (BSF, LSF, HSF) as fixed factors, and random intercepts for the participants to control for potential between-subjects differences in performance. We did not include any random slope because, as we had no reason to believe that other factors would affect the results and to keep the models as simple as possible. The analysis revealed a significant effect of spatial filtering, χ^2^ = 64.08, *p* < .001, as participants’ performance was more accurate in the BSF condition than in the HSF condition (*β*1 = 0.72, SE = 0.056, z = - 5.95, *p* < .001, 95% CI [0.64, 0.80]). There was also a significant spatial filtering x face ethnicity interaction, χ^2^ = 23.83, *p* < .001, indicating that spatial filtering affected participants’ performance differently for White and Asian faces.

**Fig 2:**
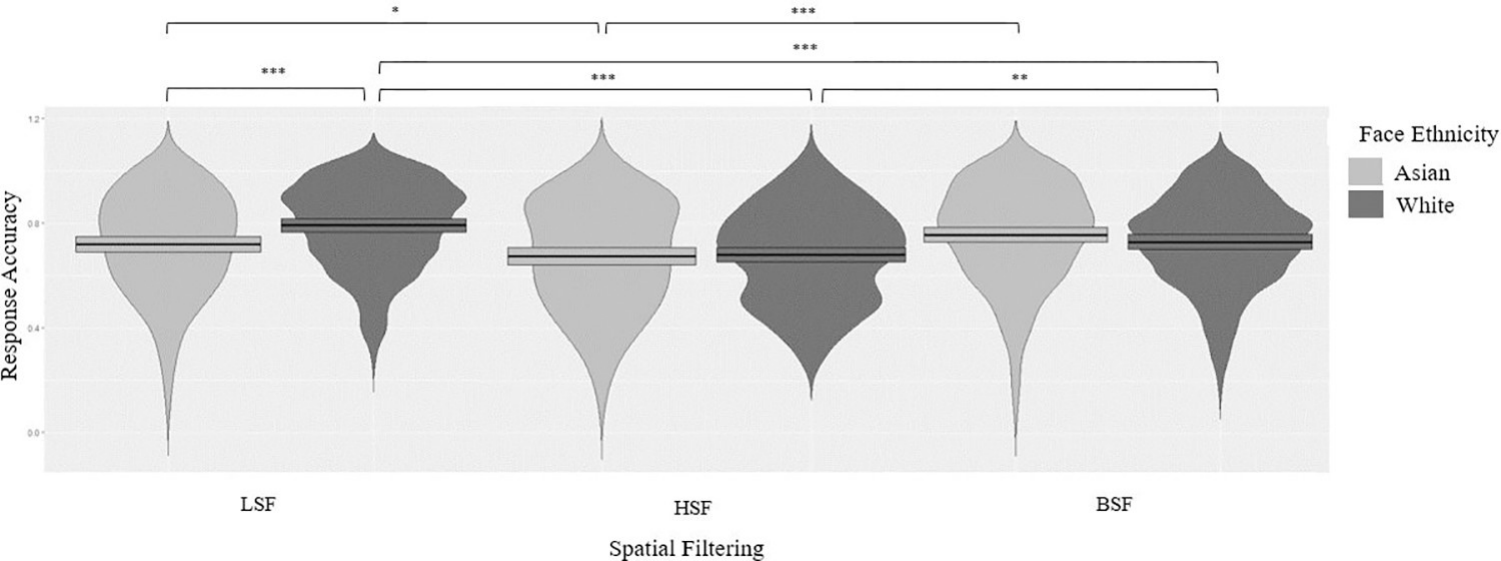
Response accuracy for European (White) and Asian faces plotted as a function of the spatial frequency condition (Broad-Spatial-Frequency, High-Spatial-Frequency, and Low-Spatial-Frequency) * *p* < .05. ** *p* < .005. *** *p* < .001.

Post-hoc comparisons (Bonferroni corrected) showed that, for White faces, participants performed more poorly in the HSF condition than in both the BSF, exp *β = 1*.*28*, *p* = .02, and the LSF conditions, exp *β = 0*.*54*, *p* < .001, and more accurately in the LSF than in the BSF condition, exp *β = 0*.*69*, *p* < .001. For Asian faces as well, participants performed more poorly in the HSF condition than in both the BSF, exp *β = 1*.*51*, *p* < 0.001, and the LSF conditions, exp *β = 0*.*79*, *p* = .046, but no other comparisons attained significance, *p*s > .342. Cross-ethnicity comparisons revealed a significant advantage in response accuracy for White over Asian faces in the LSF condition, exp *β = 0*.*67*, *p* < .001, but not in the other SF filtering conditions, *p*s > .99 (Fig 2).

### Trustworthiness scores

To obtain a measure of how consistently each face from each trustworthiness continuum was selected as the more trustworthy in the pair, participants’ response on each trial of the Pairwise Preference Task was used to compute a trustworthiness score for each face. For each subject and each trial, we gave a score of 1 to the selected face and a score of 0 to the non-selected face, and the trustworthiness scores obtained by each face were summed across trials. Since each face from each continuum appeared for a total of four times as it was paired with all the other faces of the same continuum, the related trustworthiness score could range from 0 (if never selected as most trustworthy) to 4 (if always selected as more trustworthy). Trustworthiness scores were analyzed through a Poisson regression model with three fixed factors (face ethnicity, spatial filtering, and trustworthiness intensity). Moreover, since we were interested in how face ethnicity and spatial frequency affected the slope of the relationship between trustworthiness intensity and decision outcome, we also performed the polynomial contrasts of trustworthiness intensity across the different conditions. The analysis revealed a spatial filtering main effect, χ^2^(4,8) = 9.36, p = .009, and a trustworthiness intensity main effect, χ^2^(4,8) = 1549.89, p < .001, which was qualified by significant two-way interactions between this factor and both face ethnicity, χ^2^ (4,8) = 16.00, p = 0.003, and spatial filtering, χ^2^(8,16) = 59.03, p < 0.001, and by a significant three-way interaction between face ethnicity, spatial filtering, and trustworthiness intensity, χ^2^(8,16) = 25.42, p = .001. Polynomial contrast analysis revealed that trustworthiness scores varied overall linearly across the five trustworthiness intensity steps, exp(*β*) = 2.68, z = 34.78, p < .001, but the slope was steeper in the BSF condition (exp(*β*) = 3.16, z = 21.71, p < .001) than in the HSF condition (exp(*β*) = 2.09, z = 16.91, p < .001), exp(*β*) = 0.66, z = -6.01, p < .001, with no difference between the BSF and LSF (exp(*β*)LSF = 2.91, z = 21.29, p < .001) conditions, exp(*β*) = 0.92, z = -1.09, p = .28. We found no difference between the slope for the White faces (exp(*β*) = 2.75, z = 25.50, p < .001) and for the Asian faces (exp(*β*) = 2.61, z = 23.71, p < .001), exp(*β*) = 1.05, z = 0.93, p = .35, however this difference was modulated by the spatial frequency content of the images, as it reached significance in the LSF condition (exp(*β*)White = 3.63, z = 17.09, p < .001 vs. exp(*β*)Asian = 2.34, z = 12.81, p < .001), exp(*β*) = 2.01, z = 4.79, p < .001, but not in the HSF (exp(*β*)White = 2.06, z = 11.92, p < .001 vs. exp(*β*)Asian = 2.12, z = 11.99, p < .001), exp(*β*) = 1.26, z = 1.70, p = .09, and the BSF (exp(*β*)White = 2.77, z = 14.77, p < .001, vs. exp(*β*)Asian = 3.59, z = 15.94, p < .001), exp(*β*) = 0.77, z = -2.43, p = .015, conditions.

**Fig 3:**
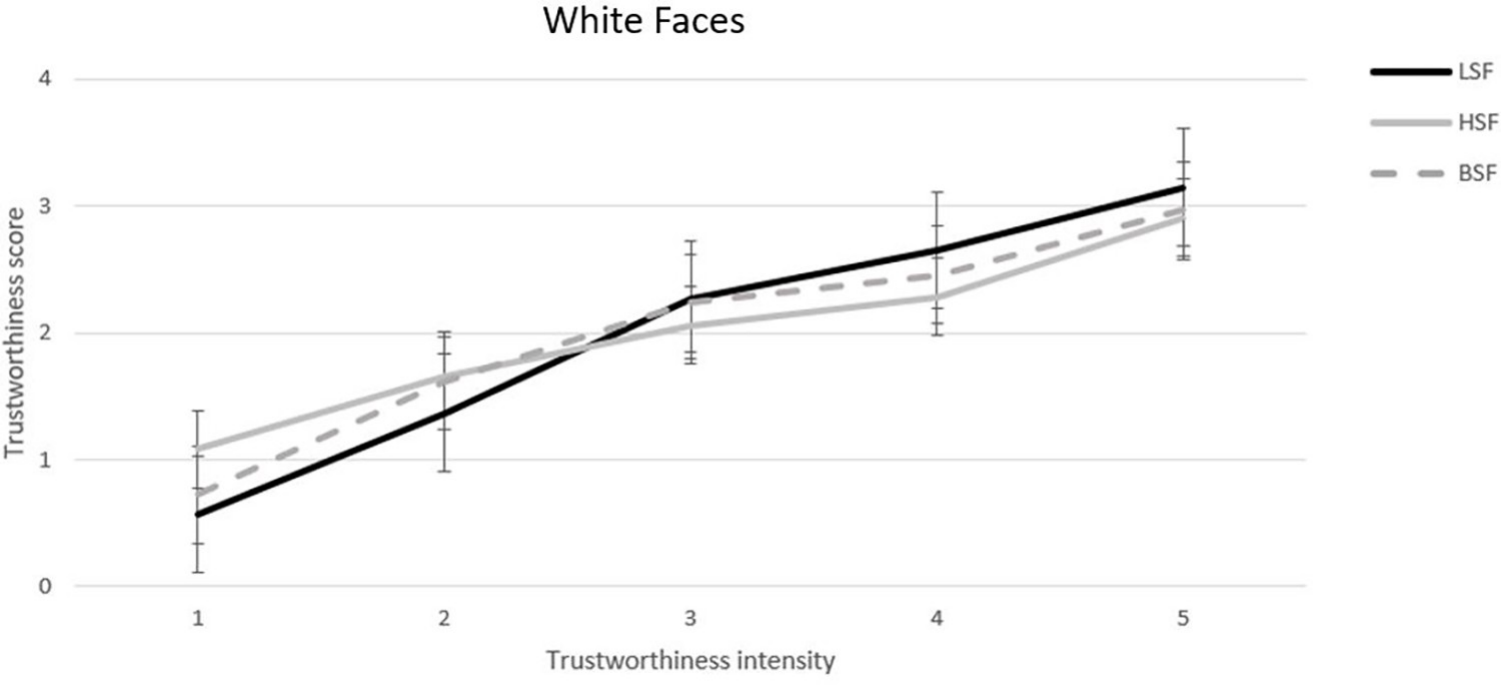
Mean trustworthiness scores for each of the five White faces composing the trustworthiness continuum in the Low-Spatial-Frequency (LSF), the High-Spatial-Frequency (HSF), and the Broad-Spatial-Frequency (BSF) conditions. Error bars refer to standard errors of the means.

To explore the impact of spatial frequency filtering within each face ethnicity, we ran two separate 3 (spatial filtering) x 5 (trustworthiness intensity) Poisson regression models, one for White faces and one for Asian faces. For White faces, trustworthiness scores varied overall linearly across the five trustworthiness intensity steps, exp(*β*) = 2.75, z = 25.50, p < .001, but the slope was steeper in the LSF condition (exp(*β*) = 3.63, z = 17.09, p < .001) than in the other two conditions (exp(*β*)HSF = 2.06, z = 11.92, p < .001; exp(*β*)BSF = 2.77, z = 14.77, p < .001), ps < .01 (Fig 3).

**Fig 4:**
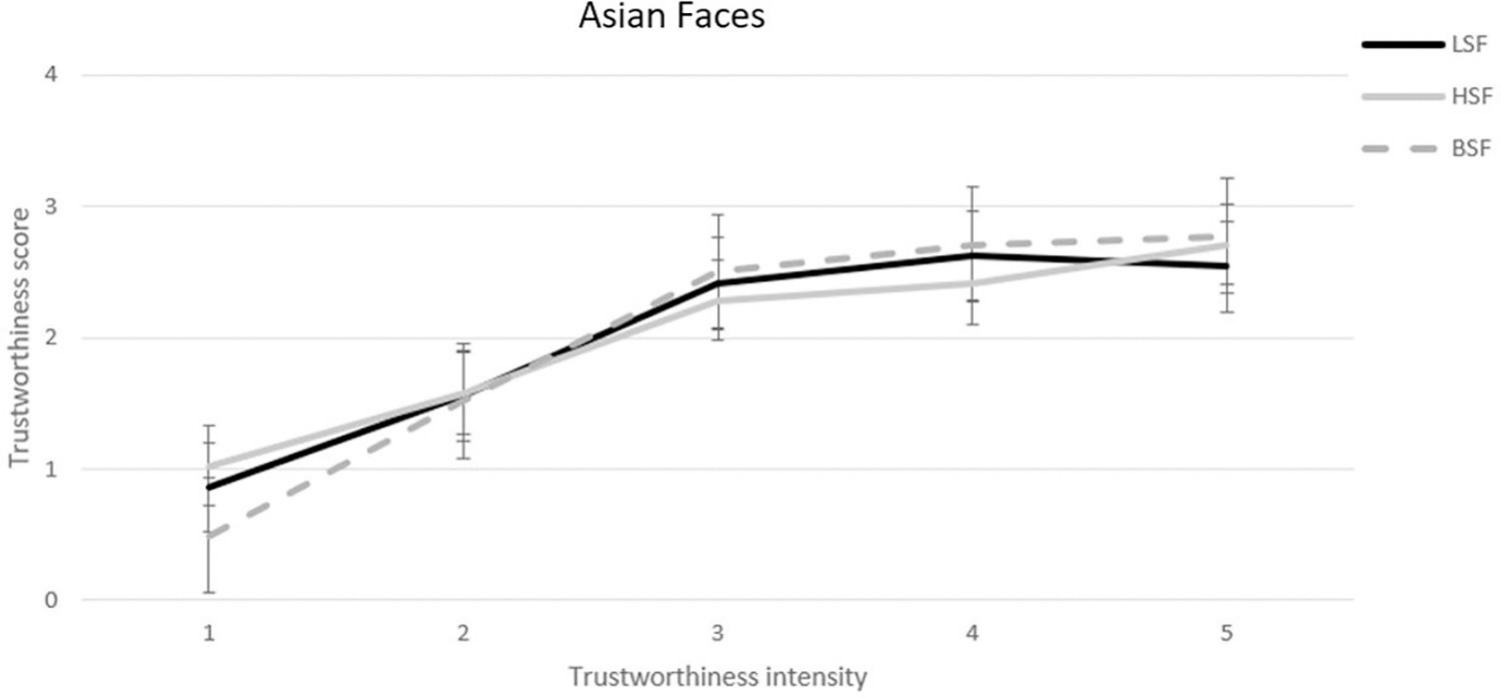
Mean trustworthiness scores for each of the five Asian faces composing the trustworthiness continuum in the Low-Spatial-Frequency (LSF), the High-Spatial-Frequency (HSF), and the Broad-Spatial-Frequency (BSF) conditions. Error bars refer to standard errors of the means.

For Asian faces the trend was overall linear across the five trustworthiness intensity steps, exp(*β*) = 2.61, z = 23.71, p < .001, but the slope was the steeper in the BSF condition (exp(*β*) = 3.59, z = 15.93, p < .001) and the gentler in the HSF condition (exp(*β*) = 2.15, z = 11.99, p < .001) with the LSF condition (exp(*β*) = 2.34, z = 12.82, p < .001) in between the two, ps < .001 (Fig 4).

## Discussion

The present study investigated the contribution of coarse and fine resolution information to trustworthiness perception in own- and other-race faces by adopting an image spatial filtering approach.

The first aim of the study was to explore the effects of spatial filtering on trustworthiness perception. Results showed that both LSF and HSF are sufficient to allow discrimination of trustworthiness intensity, but such discrimination occurs more easily (i.e., is less prone to errors) when only coarse, as compared to fine, resolution information is available in the images. Indeed, for both White and Asian faces, participants performed above chance when asked to select the more trustworthy face in a pair in all spatial filtering conditions, and the trustworthiness scores varied linearly according to the position of the faces along the continuum. This means that, irrespectively of the SF content of the images, trustworthiness intensity varied linearly for each of the five White and Asian faces as a function of the stimuli position along the trustworthiness continua.

At the same time, though, both accuracy data and trustworthiness scores also showed that LSF filtering (i.e., selective removal of the LSF content of the stimuli) had detrimental effects on participants’ performance, as participants were more accurate in the BSF condition than in the HSF condition, and the linear slope of the trustworthiness scores was steeper in the former than in the latter condition. This suggests that the detection of physical cues to trustworthiness relies more heavily on the LSF content than on the HSF content of the images. Also, this is in line with earlier evidence that emotion categorization occurs more rapidly for LSF faces than for HSF faces [26], and fits with the claim that trustworthiness judgments are overgeneralized responses to facial configurations resembling emotional expressions—i.e., the so-called emotion overgeneralization hypothesis [4, 48]. Trustworthiness judgments appear to be vehiculated by specific facial features (i.e., Action Units, see Jack & Schyns, [30]), such as up/downturned eyebrows, upward/downturned curving mouth, and a wrinkling nose, which are also involved in emotion perception. According to the emotion overgeneralization hypothesis, cues like lowered eyebrows, which, if stressed, might signal anger, would induce the perceiver to make a dispositional assumption about an otherwise emotionally neutral face, resulting in social perception of unfriendliness [2].

The contribution of LSF and HSF bands to trustworthiness perception was investigated in the current study in relation to face familiarity by testing whether spatial filtering differentially affects trustworthiness discrimination of (frequent/familiar) own-race and (infrequent/unfamiliar) other-race faces. Accuracy data confirmed earlier demonstrations that trustworthiness perception generalizes across face ethnicity [10, 11], as discrimination accuracy and for the original unfiltered faces did not differ for White and Asian faces.

Nonetheless, differential sensitivity to facial cues to trustworthiness in own-race than other-race faces was signalled in our data by the finding of greater LSF contribution to trustworthiness discrimination for White faces compared to Asian faces. Indeed, we observed a significant discrimination advantage for White over Asian faces when only LSF information was available in the stimuli. Also, although selective removal of LSF had detrimental effect on discrimination accuracy for both White and Asian faces, in the case of White faces we observed an accuracy advantage when only LSF information was available in the stimuli compared to when the entire SF bands were available in the intact BSF faces. This result was unexpected, considering that both LSF and BSF images contained the same full low spatial frequency bands. It seems to indicate that the HSF information included in the intact BSF images actually impaired trustworthiness discrimination, suggesting that detection of facial cues to trustworthiness in own-race faces is specifically mediated by the LSF content of the stimuli. This finding resonates well with previous demonstrations of greater modulation of brain activity in response to LSF emotional faces as compared to intact, unedited faces [49, 50], a finding that has been interpreted as evidence for a LSF specificity in response to emotional facial cues, which would be masked by the presence of HSF information in the intact faces.

The presence of a LSF own-race advantage in trustworthiness discrimination was also evident from the trustworthiness preference scores, whose slope was steeper for White than for Asian faces in the LSF condition, but not in the HSF and BSF conditions, while it was steeper for Asian than for White faces in the original BSF condition. Moreover, the slope was steeper in the LSF condition compared to both the HSF and BSF conditions for White faces, but not for Asian faces. Again, these findings indicate that trustworthiness perception in own-race faces was magnified when only LSF information was available in the images, and that reliance on LSF information was stronger for White than Asian faces in our European participants. These findings are in accord with those obtained by Charbonneau and colleagues [32], who showed that European-Canadian individuals with stronger implicit racial biases needed more LSF information to discriminate trustworthiness intensity in White faces compared to Black faces.

The interpretation of the current findings might be constrained by three methodological features of the study. The first relates to the online nature of the study, which limited our control over the experimental setting, with possible consequences on variability in the parameters of stimulus presentation that are relevant in relation to image spatial filtering manipulation (e.g., distance from the screen, screen luminosity, environmental lights). Although this remains true, it is important to note that, since we applied the same spatial filtering manipulations to both White and Asian faces, the impact of any intervening variable on stimulus parameters would have affected participants’ performance in the same way for both face ethnicity conditions.

A second methodological aspect that might have impacted on the results relates to the stimulus material. First, the use of morphed face images may have dampened participants’ sensitivity to differences in trustworthiness intensity in our task. Indeed, previous research has shown that absolute trustworthy judgments and discrimination accuracy are lower for morphed faces compared to real faces [51], although this is especially true for male faces, as opposed to female faces [52]. It may be that trustworthiness perception was not disrupted in our study because stimuli were female faces. However, in light of the current debate concerning the extent to which computer-generated faces are appropriate to investigate face processing expertise [53], future studies may test for the generalizability of the current results to real face images. It is indeed possible that non-morphed real face images would trigger larger face-race effects in participants’ trustworthiness discrimination with respect to those reported here in response to morphed faces.

A second aspect concerning the stimuli relates to contrast differences between our BSF, LSF and HSF faces, which might have affected participants’ perception of the filtered images, thus constraining conclusions about the mere influence of spatial frequencies on the obtained results [54]. Nonetheless, previous studies in adults [24, 43, 44] and infants [45] showed that contrast differences do not account for spatial filtering effects on discrimination of social cues from faces. Future studies might use normalized images to test whether the current findings would be replicated in the absence of any confounding between the SF and the power content of the filtered images.

A third methodological feature of the current study that might constrain the interpretation of the obtained results is the inclusion of participants from only one ethnic group. This did not allow us to investigate whether participants’ cultural background affected the source of visual information mediating trustworthiness perception. Moreover, it is often argued that the absence of a cross-cultural comparison limits the interpretation of the observed face race effects as driven by perceptual expertise for own-race faces, as those same effects might well be driven by differences in low-level stimulus characteristics rather than race per se. Cross-cultural research on face processing suggests that culture impacts the nature of visual information extracted from faces, with Westerners relying more on fine resolution information, and Easterners relying more on coarse information [55]. In light of this, future studies conducted on Easterner individuals may investigate whether the own-race LSF advantage in trustworthiness discrimination observed in the current study is a cross-cultural phenomenon or it generalizes in those individuals to other-race faces.

Overall, current demonstration of differential sensitivity to facial cues to trustworthiness in White versus Asian faces is in accord with earlier reports that trustworthiness perception differs for own-race and other-race faces in European participants [10, 12, 32]. However, unlike most of these earlier findings [10, 12] that showed a generalized positivity bias for own-race faces (i.e., own-race faces were perceived as overall more trustworthy than other-race faces), our results indicate that different perceptual processes are involved in the detection of facial configurations driving trustworthiness judgments in frequent/familiar and infrequent/unfamiliar face categories. It is well-known that perceptual expertise for over-represented faces in the individual’s social environment translates into heavier reliance on holistic/configural cues to facial identity [37, 56], and that holistic/configural processing in vision relies on relatively LSF more than on relatively HSF [57]. To the best of our knowledge, our results add to the only other existing evidence provided by Charbonneau and colleagues [32] that the SF content of the images affect trustworthiness perception differently for own-race and other-race faces in individuals with stronger implicit racial biases. Current findings extend these earlier findings to female faces and a different category of other-race faces, and generalize them to a different spatial filtering methodology, providing further evidence that the LSF-mediated holistic/configural advantage for faces from highly familiar ethnic groups in identity recognition extends also to perception of physical cues to social traits.

## References

[1] Todorov A, Said CP, Engell AD, Oosterhof NN. Understanding evaluation of faces on social dimensions. Trends In Cognitive Sciences. 2008; 12:455–460. doi: 10.1016/j.tics.2008.10.001 18951830

[2] Ames DL, Fiske ST, Todorov AT. Impression formation: A focus on others’ intents. In Decety J, Cacioppo JT (Eds.). The Oxford handbook of social neuroscience. New York: Oxford University Press. 2011; 419–433. doi: 10.1093/oxfordhb/9780195342161.013.0028

[3] Bar M, Neta M, Linz H. Very first impressions. Emotion. 2006; 6:269–278. doi: 10.1037/1528-3542.6.2.269 16768559

[4] Zebrowitz LA, Fellous JM, Mignault A, Andreoletti C. Trait impressions as overgeneralized responses to adaptively significant facial qualities: evidence from connectionist modeling. Personality And Social Psychology Review: An Official Journal Of The Society For Personality And Social Psychology. 2003; 7:194–215. doi: 10.1207/S15327957PSPR0703_01 12788687

[5] Peltola MJ, Leppänen JM, Palokangas T, Hietanen JK. Fearful faces modulate looking duration and attention disengagement in 7‐month‐old infants. Developmental science. 2008; 11:60–68. doi: 10.1111/j.1467-7687.2007.00659.x 18171368

[6] Baccolo E, Quadrelli E, Macchi Cassia V. Neural sensitivity to trustworthiness cues from realistic face images is associated with temperament: an electrophysiological study with 6-month-old infants. Social Neuroscience. 2021; 1–16. doi: 10.1080/17470919.2021.1976271 34469270

[7] Jessen S, Grossmann T. Neural and behavioral evidence for infants’ sensitivity to the trustworthiness of faces. Journal of cognitive neuroscience. 2016; 28:1728–1736. doi: 10.1162/jocn_a_00999 27315276

[8] Jessen S, Grossmann T. Neural evidence for the subliminal processing of facial trustworthiness in infancy. Neuropsychologia, 2019; 126:46–53. doi: 10.1016/j.neuropsychologia.2017.04.025 28442339

[9] Sakuta Y, Kanazawa S, Yamaguchi MK. Infants prefer a trustworthy person: An early sign of social cognition in infants. PloS one. 2018; 13. doi: 10.1371/journal.pone.0203541 30188941 PMC6126855

[10] Birkás B, Dzhelyova M, Lábadi B, Bereczkei T, Perrett DI. Cross-cultural perception of trustworthiness: The effect of ethnicity features on evaluation of faces’ observed trustworthiness across four samples. Personality and Individual Differences. 2014; 69:56–61. doi: 10.1016/j.paid.2014.05.012

[11] Xu F, Wu D, Toriyama R, Ma F, Itakura S, Lee K. Similarities and differences in Chinese and Caucasian adults’ use of facial cues for trustworthiness judgments. PLoS One. 2012; 7:4. doi: 10.1371/journal.pone.0034859 22514680 PMC3325928

[12] Sofer C, Dotsch R, Oikawa M, Oikawa H, Wigboldus DH, Todorov A. For your local eyes only: Culture-specific face typicality influences perceptions of trustworthiness. Perception. 2017; 46:914–928. doi: 10.1177/0301006617691786 28152651

[13] Over H, Cook R. Where do spontaneous first impressions of faces come from?. Cognition. 2018; 170:190–200. doi: 10.1016/j.cognition.2017.10.002 29028612

[14] Valentine T. A unified account of the effects of distinctiveness, inversion, and race in face recognition. The Quarterly Journal of Experimental Psychology Sec. A. 191; 43:161–204. doi: 10.1080/14640749108400966 1866456

[15] Scherf KS, Scott LS. Connecting developmental trajectories: Biases in face processing from infancy to adulthood. Developmental psychobiology. 2021; 54:643–663. doi: 10.1002/dev.21013 22711622

[16] Brigham JC, Malpass RS. The role of experience and contact in the recognition of faces of own‐and other‐race persons. Journal of social issues. 1985; 41:139–155. doi: 10.1111/j.1540-4560.1985.tb01133

[17] Lebrecht S, Pierce LJ, Tarr MJ, Tanaka JW. Perceptual other-race training reduces implicit racial bias. PLoS One. 2009; 4:e4215. doi: 10.1371/journal.pone.0004215 19156226 PMC2627769

[18] Young SG, Hugenberg K, Bernstein MJ, Sacco DF. Perception and motivation in face recognition: A critical review of theories of the cross-race effect. Personality and Social Psychology Review. 2012; 16:116–142. doi: 10.1177/1088868311418987 21878608

[19] Rhodes G, Hayward WG, Winkler C. Expert face coding: configural and component coding of own-race and other-race faces. Psychonomic bulletin & review. 2006; 13:499–505. doi: 10.3758/bf03193876 17048737

[20] Tanaka JW, Kiefer M, Bukach CM. A holistic account of the own-race effect in face recognition: Evidence from a cross-cultural study. Cognition. 2004; 93:B1–B9. doi: 10.1016/j.cognition.2003.09.011 15110726

[21] Short LA, Hatry AJ, Mondloch CJ. The development of norm-based coding and race-specific face prototypes: An examination of 5-and 8-year-olds’ face space. Journal of Experimental Child Psychology. 2011; 108:338–357. doi: 10.1016/j.jecp.2010.07.007 20822777

[22] Goffaux V, Hault B, Michel C, Vuong QC, Rossion B. The respective role of low and high spatial frequencies in supporting configural and featural processing of faces. Perception. 2005;34: 77–86. doi: 10.1068/p5370 15773608

[23] Goffaux V, Rossion B. Faces are “spatial”-Holistic face perception is supported by low spatial frequencies. Journal of Experimental Psychology: Human Perception and Performance. 2006;32: 1023–1039. doi: 10.1037/0096-1523.32.4.1023 16846295

[24] Vlamings PHJM, Goffaux V, Kemner C. Is the early modulation of brain activity by fearful facial expressions primarily mediated by coarse low spatial frequency information? Journal of Vision. 2009; 9:1–13. doi: 10.1167/9.5.12 19757890

[25] Vuilleumier P, Armony JL, Driver J, Dolan RJ. Distinct spatial frequency sensitivities for processing faces and emotional expressions. Nature Neurosciences. 2003; 6:624–631. doi: 10.1038/nn1057 12740580

[26] Schyns P, Oliva A. Angry Dr. and Mr. Smile: when categorization flexible modifies the perception of faces in rapid visual presentations. Cognition. 1999; 69:243–265. doi: 10.1016/S0010-0277(98)00069-9 10193048

[27] Holmes A, Green S, Vuilleumier P. The involvement of distinct visual channels in rapid attention towards fearful facial expressions. Cognition & Emotion. 2005; 19:899–922. doi: 10.1080/02699930441000454

[28] Derrington AM, Lennie P. Spatial and temporal contrast sensitivities of neurones in Lateral Geniculate Nucleus of Macaque. Journal of Physiology. 1984;357:219–240. doi: 10.1113/jphysiol.1984.sp015498 6512690 PMC1193256

[29] Skottun BC. On the use of spatial frequency to isolate contributions from the magnocellular and parvocellular systems and the dorsal and ventral cortical streams. Neuroscience and Biobehavioral Reviews. 2015; 56:266–275. doi: 10.1016/j.neubiorev.2015.07.002 26188134

[30] Jack RE, Schyns PG. The human face as a dynamic tool for social communication. Current Biology. 2015; 25: R621–R634. doi: 10.1016/j.cub.2015.05.052 26196493

[31] Dotsch R, Todorov A. Reverse Correlating Social Face Perception. Social Psychological and Personality Science. 2012; 3, 562–571. 10.1177/1948550611430272.

[32] Charbonneau I, Robinson K, Blais C, Fiset D. Implicit race attitudes modulate visual information extraction for trustworthiness judgments. Plos one. 2020;15:9, e0239305. doi: 10.1371/journal.pone.0239305 32970725 PMC7514083

[33] Robinson K, Blais C, Duncan J, Forget H, Fiset D. The dual nature of the human face: there is a little Jekyll and a little Hyde in all of us. Frontiers in psychology. 2014; 5, 139. doi: 10.3389/fpsyg.2014.00139 24639658 PMC3944200

[34] Said CP, Baron SG, Todorov A. Nonlinear amygdala response to face trustworthiness: contributions of high and low spatial frequency information. Journal of Cognitive Neuroscience. 2009; 21:519–528. doi: 10.1162/jocn.2009.21041 18564045

[35] Gosselin F, Schyns PG. Bubbles: a technique to reveal the use of information in recognition tasks. Vision research. 2001;41:2261–2271. doi: 10.1016/s0042-6989(01)00097-9 11448718

[36] Oosterhof NN, Todorov A. The functional basis of face evaluation. Proceedings of the National Academy of Sciences. 2008; 105:11087–11092. doi: 10.1073/pnas.0805664105 18685089 PMC2516255

[37] Michel C, Rossion B, Han J, Chung CS, Caldara R. Holistic processing is finely tuned for faces of one’s own race. Psychological Science. 2006; 17:608–615. doi: 10.1111/j.1467-9280.2006.01752.x 16866747

[38] Baccolo E, Macchi Cassia V. Age‐Related Differences in Sensitivity to Facial Trustworthiness: Perceptual Representation and the Role of Emotional Development. Child Development. 2020; 91: 1529–1547. doi: 10.1111/cdev.13340 31769004

[39] Green P, MacLeod CJ. SIMR: an R package for power analysis of generalized linear mixed models by simulation. Methods in Ecology and Evolution. 2016;7:493–498.

[40] DeBruine L. Webmorph (Version v0.0.0.9001). Zenodo. 2017. doi: 10.5281/zenodo.1073696

[41] Ma DS, Correll J, Wittenbrink B. The Chicago face database: A free stimulus set of faces and norming data. Behavior Research Methods. 2015; 47(4):1122–1135. doi: 10.3758/s13428-014-0532-5 25582810

[42] Dobkins KR, Harms R. The face inversion effect in infants is driven by high, and not low, spatial frequencies. Journal of Vision. 2014;14:1–1. 10.1167/14.1.1. PMC3880109 24385345

[43] Harel A, Bentin S. Stimulus type, level of categorization, and spatial-frequencies utilization: Implications for perceptual categorization hierarchies. Journal of Experimental Psychology: Human Perception and Performance. 2009; 35:1264–1273. doi: 10.1037/a0013621 19653764 PMC2800924

[44] Munsters NM, van den Boomen C, Hooge IT, Kemner C. The role of global and local visual information during gaze-cued orienting of attention. PloS one. 2016; 11:e0160405. doi: 10.1371/journal.pone.0160405 27560368 PMC4999176

[45] van den Boomen C, Munsters NM, Kemner C. Emotion processing in the infant brain: The importance of local information. Neuropsychologia. 2009; 126:62–68. doi: 10.1016/j.neuropsychologia.2017.09.006 28889996

[46] Jessen S, Grossmann T. Exploring the role of spatial frequency information during neural emotion processing in human infants. Frontiers in human neuroscience. 2017; 11:1–8. doi: 10.3389/fnhum.2017.00486 29062275 PMC5640713

[47] Meteyard L, Davies RAI. Best practice guidance for linear mixed effects models in psychological science. J. Memory Language. 2020; 112:104092. doi: 10.1016/j.jml.2020.104092

[48] Said CP, Sebe N, Todorov A. Structural resemblance to emotional expressions predicts evaluation of emotionally neutral faces. Emotion. 2009; 9:260–264. doi: 10.1037/a0014681 19348537

[49] Carretié L, Hinojosa JA, López-Martín S, Tapia M. An electrophysiological study on the interaction between emotional content and spatial frequency of visual stimuli. Neuropsychologia. 2007; 45:1187–1195. doi: 10.1016/j.neuropsychologia.2006.10.013 17118408

[50] Maratos FA, Mogg K, Bradley BP, Rippon G, Senior C. Coarse threat images reveal theta oscillations in theamygdala: a magnetoencephalography study. Cogn. Affect.Behav. Neurosci. 2009; 9:133–143. doi: 10.3758/CABN.9.2.133 19403890

[51] Balas B, Pacella J. Trustworthiness perception is disrupted in artificial faces. Computers in Human Behavior. 2017; 77: 240–248. doi: 10.1016/j.chb.2017.08.045

[52] Balas B, Tupa L, Pacella J. Measuring social variables in real and artificial faces. Computers in Human Behavior. 2018; 88, 236–243.

[53] Crookes K, Ewing L, Gildenhuys JD, Kloth N, Hayward WG, Oxner M, Pond S, Rhodes G. How well do computer-generated faces tap face expertise?. PloS one. 2015; 10:11, e0141353. doi: 10.1371/journal.pone.0141353 26535910 PMC4633121

[54] Perfetto S, Wilder J, Walther DB. Effects of spatial frequency filtering choices on the perception of filtered images. Vision. 2020; 4:2–29. doi: 10.3390/vision4020029 32466442 PMC7355859

[55] Tardif J, Fiset D, Zhang Y, Estéphan A, Cai Q, Luo C, et al. Culture shapes spatial frequency tuning for face identification. Journal of Experimental Psychology: Human Perception and Performance. 2017; 43: 294. doi: 10.1037/xhp0000288 27819456

[56] Yao Q, Zhao L. Using spatial frequency scales for processing own-race and other-race faces: An ERP analysis. Neurosci Lett. 2019; 13. 705:167–171. doi: 10.1016/j.neulet.2019.04.059 31051221

[57] Lamb MR, Yund EW. The role of spatial frequency in the processing of hierarchically organized stimuli. Perception & Psychophysics. 1993; 54:773–784. doi: 10.3758/bf03211802 8134247

